# Association between the composite dietary antioxidant index and the prevalence and recurrence of kidney stones: results of a nationwide survey

**DOI:** 10.3389/fnut.2024.1413937

**Published:** 2024-06-19

**Authors:** Huan Zhu, Yinmei Chen, Yue Feng, Hui Chen

**Affiliations:** Department of Urology, Harbin Medical University Cancer Hospital, Harbin, China

**Keywords:** composite dietary antioxidant index, NHANES, kidney stones, kidney stones recurrence, cross-sectional study

## Abstract

**Aim:**

This study aims to evaluate the relationship between the Composite Dietary Antioxidant Index (CDAI) and the prevalence and recurrence of kidney stones.

**Methods:**

Data from the National Health and Nutrition Examination Survey (NHANES) collected between 2007 and 2014 were used in this cross-sectional analysis. The CDAI was derived by standardizing the intake of dietary antioxidants from 24 h dietary recalls. The study assessed the prevalence and recurrence of kidney stones based on questionnaire responses. The association between the CDAI and both the prevalence and recurrence of kidney stones was investigated using multivariable logistic regression. Subgroup analyses and interaction tests further evaluated the robustness of this relationship.

**Results:**

The study included 20,743 participants, and the reported incidence and recurrence rates of kidney stones were 9.09 and 2.90%, respectively. After stratifying the CDAI into tertiles, an inverse trend was observed in both kidney stones’ prevalence and recurrence probabilities with increasing CDAI levels. Adjusting for confounding factors, individuals in the top tertile had a 23% lower prevalence of kidney stones (OR = 0.77, 95% CI: 0.66, 0.90, *p* = 0.0011) and a 39% lower recurrence rate (OR = 0.61, 95% CI: 0.47, 0.80, *p* = 0.0003) than those in the bottom tertile. In addition, interaction tests showed that age, gender, body mass index, hypertension, and diabetes did not significantly affect the relationship between CDAI levels and kidney stone prevalence and recurrence rates.

**Conclusion:**

Our study suggests that increased levels of CDAI are associated with reduced incidence and recurrence rates of kidney stones. Therefore, increasing the intake of dietary antioxidants may be an effective strategy for preventing kidney stones and their recurrence.

## 1 Introduction

Kidney stones, hard deposits of minerals and salts in urine, cause significant patient discomfort and impose a considerable economic burden on public health systems. In the United States, the annual expenditure on treating kidney stones is estimated to be approximately 10 billion dollars (1). The prevalence of kidney stones exhibits notable regional variations: 7–13% in North America, 5–9% in Europe, and is relatively lower in some areas of Asia (2). Absent preventive measures, it is estimated that approximately 50% of patients may experience a recurrence of kidney stones within 5 years (3). Recent studies have elucidated a close association between kidney stones and several health issues, such as hypertension, chronic kidney disease, and end-stage renal disease (4, 5). The increasing incidence of kidney stones, driven by global lifestyle and dietary habits shifts, underscores the urgency of implementing effective early prevention strategies (6, 7). Such strategies include maintaining a balanced fluid intake and adjusting dietary habits to mitigate the risk of kidney stone formation (8).

Antioxidants are substances that can prevent or slow down cell damage, including vitamin A, vitamin E, β-carotene, and various phytochemicals found in many foods (9). They neutralize free radicals in the body, reducing oxidative stress and releasing inflammatory mediators (10, 11). Through its metabolic processes, vitamin A can regulate the pH of the urine, keeping calcium oxalate crystals in a dispersed state and facilitating their excretion (12). Existing research suggests that the antioxidant selenium can reduce oxidative stress in the urine, inhibiting the tendency for calcium oxalate crystals to form and, consequently, reducing the incidence of kidney stones (13). Diets rich in antioxidants are associated with a lower incidence of kidney stones (14).

Recent research has started to explore the potential connection between antioxidants and kidney stone formation. However, the results have been inconsistent. For instance, an *in vitro* study using LLC-PK1 cell cultures found that vitamin C could reduce oxalate-induced oxidative renal damage and calcium oxalate crystal deposition (15). Conversely, an animal study involving male Wistar rats demonstrated that, despite being an effective antioxidant, vitamin C did not reduce oxidative stress-related damage associated with calcium oxalate (16). These findings suggest that the role of individual antioxidants may be limited, highlighting the need for a more comprehensive approach to antioxidant assessment. The Composite Dietary Antioxidant Index (CDAI) serves as an indicator for assessing the overall antioxidant capacity of the human body, encompassing vitamins A, C, and E, zinc, selenium, and carotenoids. It offers a thorough assessment of a diet’s capacity to mitigate oxidative stress and neutralize free radicals (17). Previous research has demonstrated the ability of the CDAI to improve outcomes in conditions such as heart failure, hypertension, depression, and atherosclerotic cardiovascular disease, as well as reducing the risk of these conditions (18–21). However, the relationship between CDAI and both the occurrence and recurrence of kidney stones has not yet been investigated.

In this study, we analyzed data from the 2007–2014 National Health and Nutrition Examination Survey (NHANES) to investigate the association between CDAI and the prevalence and recurrence of kidney stones.

## 2 Materials and methods

### 2.1 Study population

**Figure 1:**
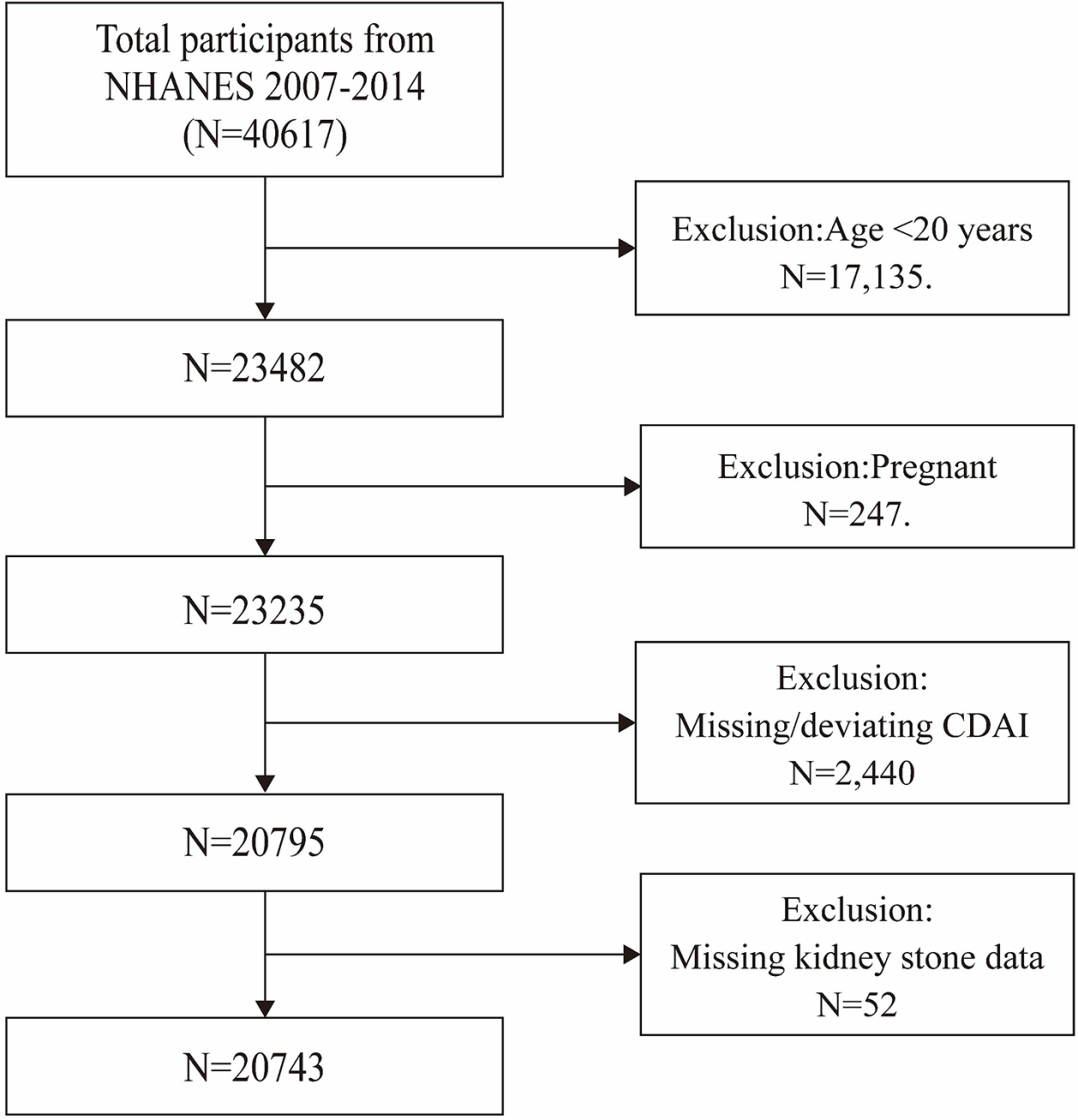
Flow chart of participants selection. NHANES, National Health and Nutrition Examination Survey.

Data for this investigation were drawn exclusively from NHANES, a cross-sectional survey that conducts interviews and physical examinations on a randomly selected, nationally representative cohort to provide a comprehensive assessment of the health and nutritional status of the American population (22). The study’s methodology received formal approval from the National Center for Health Statistics (NCHS). Written informed consent was obtained from all participants. The study initially enrolled 46,017 participants over four consecutive cycles from 2007 to 2014, with complete data on CDAI, kidney stones, and their recurrence only available within these cycles. After excluding people aged under 20 (*n* = 23,482), pregnant women (*n* = 247), participants with incomplete CDAI data (*n* = 2,440), and those without kidney stone data (*n* = 52), a total of 20,743 eligible participants were retained for the final analysis (Figure 1).

### 2.2 Assessment of composite dietary antioxidant index

The dietary recall interviews in NHANES were conducted face-to-face by trained dietary interviewers. These interviews follow a set of standardized guidelines for the nutritional assessment of each food item consumed. The first dietary recall interview takes place at the Mobile Examination Center (MEC), followed by a telephone interview occurring 3 to 10 days afterward. Throughout the data collection process, rigorous checks are made to address any omissions or inconsistencies in the reports and ensure the interview completeness (23). The calculation of the CDAI includes six major antioxidant components: vitamins A, C, E, zinc, selenium, and carotenoids. The sources of these dietary antioxidants are strictly limited to food intake, explicitly excluding any contribution from nutritional supplements, pharmaceuticals, or antioxidants in drinking water. The CDAI score is derived by deducting the average intake of each antioxidant from the individual’s intake, dividing this by the standard deviation, and subsequently aggregating the standardized scores for all antioxidant components.


$$\mathrm{CDAI}=\overset{6}{\underset{i=1}{\sum }}\frac{\left(\mathrm{Antioxidant}\;\mathrm{intak}e_{i}-mean_{i}\right)}{SD_{i}}$$

### 2.3 Diagnosis of kidney stones

In our selected cycles of NHANES, data regarding kidney stones and their expulsion were gathered at participants’ residences through the Computer-Assisted Personal Interviewing (CAPI) system by extensively trained interviewers. The prevalence of kidney stones was determined by answering two key questions: “Have you ever had kidney stones?” and “How many times have you had kidney stones?” Participants who reported having had kidney stones in response to the first question were classified as having had kidney stones. Those who reported passing kidney stones twice or more in response to the second question were categorized as having recurrent kidney stones.

### 2.4 Covariables

The covariates in this study include demographic and health-related variables, such as age (measured in years); sex (male/female); race (Mexican American, Other Hispanic, Non-Hispanic White, Non-Hispanic Black, and Other); educational attainment (less than high school, high school or GED equivalent, more than high school, and other); the poverty income ratio (PIR); the body mass index (BMI); serum levels of calcium, phosphorus, total cholesterol, and triglycerides; the presence of hypertension and diabetes; as well as intake of total energy, alcohol, and water. Hypertension was identified through either self-reported diagnosis, systolic blood pressure readings ≥140 mmHg, diastolic measurements ≥90 mmHg, or the use of antihypertensive medication (24). Diabetes mellitus was identified based on one or more of the following: clinical diagnosis, use of glucose-lowering medication, oral glucose tolerance test (OGTT) results ≥200 mg/dL, hemoglobin A1c (HbA1c) levels ≥6.5%, or fasting plasma glucose levels ≥126 mg/dL (25).

### 2.5 Statistical analysis

Statistical analyses in this study were performed using R (version 4.2.0) and EmpowerStats (version 4.2) with a statistical significance threshold of *p* < 0.05. In the descriptive analysis, continuous variables were analyzed using *t*-tests and presented with means and standard deviations, while categorical variables were evaluated with chi-squared tests and expressed as percentages. The CDAI was categorized into tertiles and evaluated using multivariable logistic regression to assess its association with the prevalence and recurrence of kidney stones across three distinct models. Model 1 served as the base model without any adjustments for covariates. Model 2, minimally adjusted, considered age, gender, and ethnicity. Model 3 included adjustments for a comprehensive set of covariates: age, gender, race, educational level, PIR, BMI, total energy intake, total alcohol intake, total water intake, serum creatinine levels, total cholesterol, triglycerides, serum calcium, serum phosphorus, hypertension, and diabetes mellitus status. Additionally, stratified analyses and interaction tests focused on age, gender, BMI, hypertension, and diabetes were conducted to explore further the relationship between CDAI and kidney stone prevalence and recurrence among various demographic groups.

## 3 Results

### 3.1 Baseline characteristics

Table 1 outlines the demographic characteristics of 20,743 individuals, detailing a mean age of 49.59 ± 17.66 years, with 49.33% males and 50.67% females. The CDAI was divided into tertiles (tertile 1: −8.29 to 1.55; tertile 2: −1.54 to 1.53; tertile 3: 1.54 to 48.66). In the highest tertile, participants were generally younger (48.06 ± 17.15 years), predominantly male (63.70%), chiefly non-Hispanic white (48.15%), and more often college-educated (60.25% with a degree). They exhibited a greater income-to-poverty ratio (2.76 ± 1.62) when compared with the lowest tertile. Additionally, the prevalence of hypertension and diabetes was less in T3 (38.60 and 15.09%, respectively) than in T1 (47.19 and 21.25%) (Table 1).

**Table 1:** Basic characteristics of participants by composite dietary antioxidant index among U.S. adults.

Variables	Composite dietary antioxidant index				*p*-value
	Total	T1 (−8.29–1.55)	T2 (−1.54–1.53)	T3 (1.54–48.66)	
*N*	20,743	6,912	6,911	6,920	
Age (years)	49.59 ± 17.66	51.01 ± 18.06	49.70 ± 17.65	48.06 ± 17.15	<0.001
Gender, (%)					<0.001
Male	10,232 (49.33%)	2,531 (36.62%)	3,293 (47.65%)	4,408 (63.70%)	
Female	10,511 (50.67%)	4,381 (63.38%)	3,618 (52.35%)	2,512 (36.30%)	
Races, (%)					<0.001
Mexican American	3,069 (14.80%)	1,076 (15.57%)	1,013 (14.66%)	980 (14.16%)	
Other Hispanic	2068 (9.97%)	756 (10.94%)	715 (10.35%)	597 (8.63%)	
Non-Hispanic White	9,317 (44.92%)	2,849 (41.22%)	3,136 (45.38%)	3,332 (48.15%)	
Non-Hispanic Black	4,378 (21.11%)	1,688 (24.42%)	1,389 (20.10%)	1,301 (18.80%)	
Other Races	1911 (9.21%)	543 (7.86%)	658 (9.52%)	710 (10.26%)	
Educational levels, (%)					<0.001
< high school	5,354 (25.81%)	2,383 (34.48%)	1,678 (24.28%)	1,293 (18.68%)	
High school or GED	4,742 (22.86%)	1719 (24.87%)	1,569 (22.70%)	1,454 (21.01%)	
> high school	10,624 (51.22%)	2,799 (40.49%)	3,656 (52.90%)	4,169 (60.25%)	
Others	23 (0.11%)	11 (0.16%)	8 (0.12%)	4 (0.06%)	
PIR	2.49 ± 1.57	2.14 ± 1.45	2.56 ± 1.58	2.76 ± 1.62	<0.001
BMI, (%)					<0.001
Normal weight	6,041 (29.12%)	1910 (27.63%)	1950 (28.22%)	2,181 (31.52%)	
Overweight	6,898 (33.25%)	2,232 (32.29%)	2,297 (33.24%)	2,369 (34.23%)	
Obesity	7,804 (37.62%)	2,770 (40.08%)	2,664 (38.55%)	2,370 (34.25%)	
Total energy (kcal)	2036.66 ± 867.46	1431.25 ± 497.12	1987.50 ± 553.24	2690.48 ± 952.47	<0.001
Total alcohol intake (gm)	8.62 ± 22.73	6.69 ± 20.99	8.49 ± 21.76	10.67 ± 25.07	<0.001
Total water drank (gm)	979.46 ± 963.57	848.11 ± 903.79	967.88 ± 903.94	1122.22 ± 1055.32	<0.001
Serum creatinine (mg/dL)	0.91 ± 0.45	0.92 ± 0.58	0.90 ± 0.42	0.91 ± 0.31	0.022
Serum calcium (mmol/L)	2.36 ± 0.09	2.35 ± 0.09	2.36 ± 0.09	2.36 ± 0.09	0.022
Serum phosphorus (mmol/L)	1.21 ± 0.18	1.21 ± 0.18	1.21 ± 0.18	1.21 ± 0.18	0.660
Triglyceride (mmol/L)	1.49 ± 1.01	1.49 ± 0.83	1.49 ± 1.16	1.48 ± 1.01	0.880
Total cholesterol (mmol/L)	5.01 ± 1.06	5.04 ± 1.07	5.02 ± 1.06	4.97 ± 1.04	<0.001
Hypertension, (%)	8,845 (42.64%)	3,262 (47.19%)	2,912 (42.14%)	2,671 (38.60%)	<0.001
Diabetes, (%)	3,756 (18.11%)	1,469 (21.25%)	1,243 (17.99%)	1,044 (15.09%)	<0.001
Kidney stone, (%)	1886 (9.09%)	658 (9.52%)	637 (9.22%)	591 (8.54%)	0.017
Kidney stone recurrence, (%)	601 (2.90%)	215 (3.11%)	214 (3.10%)	172 (2.49%)	0.010

Continuous variables were showed as mean ± SE, categorical variables were showed as frequency (percentage). PIR, poverty income ratio; BMI, body mass index; GED, general educational development.

### 3.2 Association of CDAI with kidney stones

Table 2 illustrates how CDAI correlates with kidney stone cases across three progressively adjusted models. There’s a marked negative link between CDAI levels and the prevalence of kidney stones. Complete adjustments reveal that a unit increase in CDAI correlates with a 3% lower prevalence of kidney stones (OR = 0.97; 95% CI: 0.95–0.98). Sensitivity analyses segmented CDAI into tertiles, showing in the fully adjusted scenario that individuals in the top CDAI tertile (T3) experienced a 23% lower chance of kidney stones compared to the bottom tertile (T1) (OR = 0.77; 95% CI: 0.66–0.90, *p* = 0.0011).

**Table 2:** Association between the composite dietary antioxidant index and kidney stones and kidney stone recurrence.

Variables	Model 1		Model 2		Model 3	
	OR (95%CI)	*p*-value	OR (95%CI)	*p*-value	OR (95%CI)	*p*-value
**Kidney stone**						
CDAI	0.99 (0.97, 1.00)	0.018	0.98 (0.97, 0.99)	0.002	0.97 (0.95, 0.98)	<0.001
**CDAI tertile**						
tertile 1	1		1		1	
tertile 2	0.96 (0.86, 1.08)	0.542	0.92 (0.82, 1.04)	0.189	0.89 (0.79, 1.01)	0.081
tertile 3	0.89 (0.79, 1.00)	0.045	0.83 (0.74, 0.94)	0.003	0.77 (0.66, 0.90)	0.001
P for trend		0.042		0.003		0.001
**Kidney stone recurrence**						
CDAI	0.97 (0.95, 0.99)	0.011	0.96 (0.94, 0.98)	<0.001	0.93 (0.90, 0.96)	<0.001
**CDAI tertile**						
tertile 1	1		1		1	
tertile 2	1.00 (0.82, 1.21)	0.962	0.91 (0.75, 1.10)	0.320	0.86 (0.70, 1.07)	0.174
tertile 3	0.79 (0.65, 0.97)	0.026	0.68 (0.55, 0.84)	<0.001	0.61 (0.47, 0.80)	<0.001
P for trend		0.022		<0.001		<0.001

Model 1: no covariates were adjusted.

Model 2: age, gender, and race were adjusted.

Model 3: age, gender, race, educational level, PIR, BMI, total energy intake, total alcohol intake, total water intake, serum creatinine levels, total cholesterol, triglycerides, serum calcium, serum phosphorus, hypertension, diabetes were adjusted.

PIR, poverty income ratio; BMI, body mass index; GED, general educational development.

### 3.3 Association between CDAI and recurrence of kidney stones

A significant negative association with CDAI was observed for kidney stone recurrence. Within the fully adjusted model, each unit increase in CDAI correlated with a 7% decrease in the recurrence odds (OR = 0.93; 95% CI: 0.90–0.96). Dividing CDAI into tertiles, the analysis indicated that participants in the upper tertile had 39% lower odds of experiencing recurrent kidney stones compared to those in the lowest tertile (OR = 0.61; 95% CI: 0.47–0.80) (Table 2).

### 3.4 Subgroup analyses

**Figure 2:**
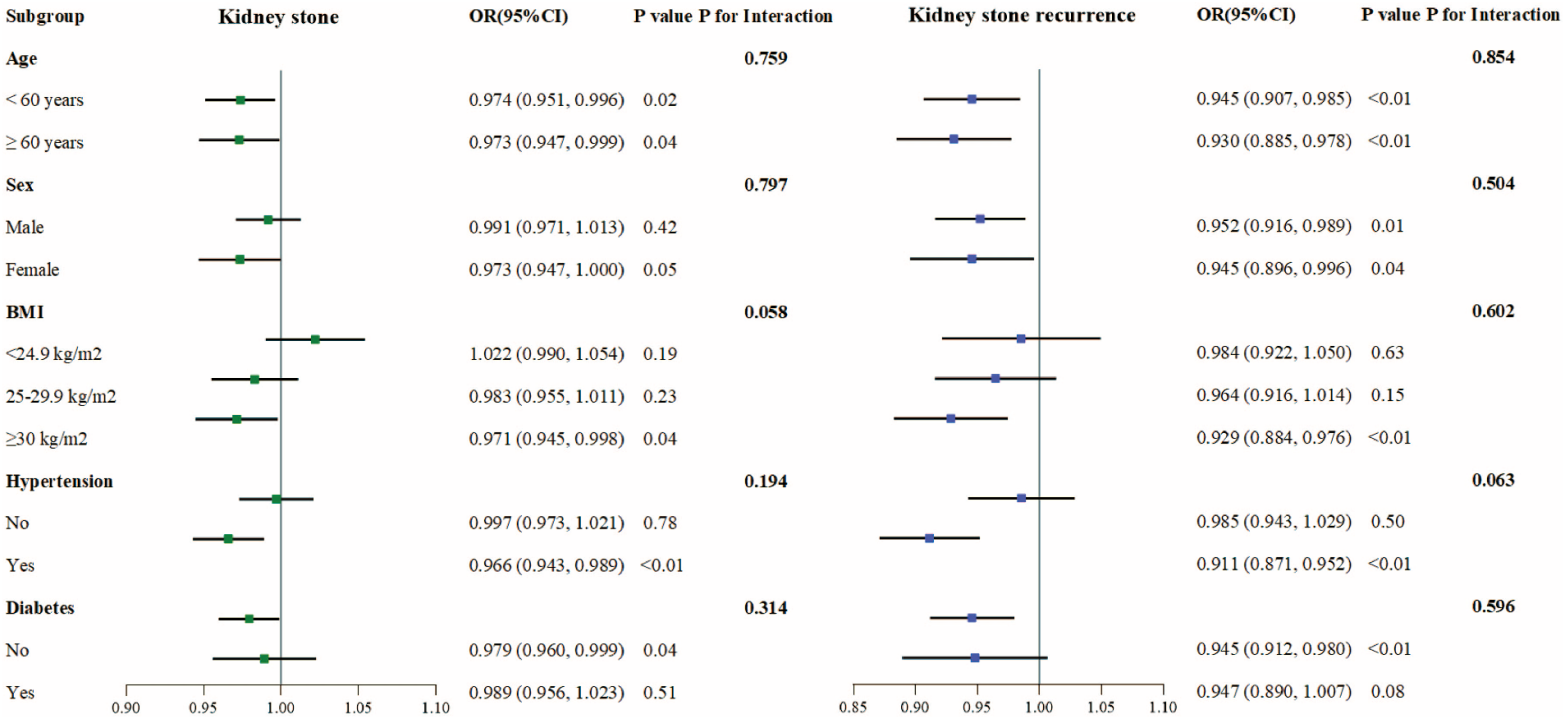
Subgroup analysis of the association between CDAI and prevalence and recurrence of kidney stones.

To evaluate the consistency of the link between CDAI and kidney stone occurrence, subgroup analyses and interaction tests were conducted considering variables such as age, sex, BMI, hypertension, and diabetes status. A consistent negative correlation between CDAI and kidney stone occurrence was observed without significant variations across different subgroups, as interaction effects were absent (*p* > 0.05) (Figure 2).

For kidney stone recurrence, similar subgroup analyses reaffirmed the uniform persistence of the inverse correlation with CDAI across stratified groups, with no significant interactions detected (*p* > 0.05), implying that the association between CDAI and stone recurrence was stable across the variables analyzed (Figure 2).

## 4 Discussion

In our cross-sectional analysis of 20,743 individuals, we observed that higher CDAI levels were inversely correlated with both the prevalence and recurrence of kidney stones. Further subgroup analyses and tests for interaction effects showed that this association did not change with variations in age, sex, BMI, hypertension, or diabetes status. This indicates an association between higher CDAI levels and a lower prevalence and recurrence of kidney stones. These findings highlight a potential approach for clinical practice: modifying dietary patterns to enhance antioxidant consumption could correlate with reduced prevalence and recurrence of kidney stones.

This research is the first to examine the association between the levels of CDAI and the prevalence and recurrence of kidney stones. Earlier studies have predominantly focused on the correlation between specific antioxidants and kidney stones. For example, in a case–control study with 75 kidney stone patients, Kato observed that the mean plasma concentrations of vitamins A and E in these patients (vitamin A: 13.18 ± 7.95 mg/d; vitamin E: 0.66 ± 0.23 mg/d) were notably lower compared to those in the healthy controls (vitamin A: 34.99 ± 11.40 mg/d; vitamin E: 1.10 ± 0.23 mg/d) (26). Similarly, in another case–control study involving 104 individuals with calcium oxalate stones, Atakan et al. found that urinary zinc and magnesium levels were significantly elevated in the healthy controls compared to the stone-forming group (*p* < 0.0001). This implies that zinc and magnesium may play a role in preventing the formation of calcium oxalate stones (27). Furthermore, recent research suggests that diets rich in antioxidants and certain dietary habits may be linked to a reduced incidence of kidney stones. For instance, Ilbey et al. demonstrated that administering pomegranate juice to rats with ethylene glycol (EG)-induced hyperoxaluria lowered calcium oxalate stone formation by reducing the expression of ROS, NF-kB, and p38-MAPK, thereby inhibiting oxidative stress (28). In a longitudinal study spanning three large cohorts, Rodrigue found that participants with higher adherence to the Mediterranean diet had a 13–41% reduced risk of developing kidney stones compared to those with lower adherence (29). Nevertheless, findings on the relationship between antioxidants and kidney stones have been mixed; for instance, a prospective study across three sizable cohorts noted no link between vitamin B6 intake and kidney stone occurrence (30). Jian et al. found no association between dietary antioxidants and kidney stones in a cross-sectional study (31). In contrast, Lin’s research using the NHANES database showed that the Oxidative Balance Score (OBS) could be a significant predictor for kidney stones (32). This discrepancy may stem from focusing solely on the effects of individual antioxidants on kidney stone formation, thereby neglecting potential interactions and synergistic effects among antioxidants. Moludi found in a cohort study that total dietary antioxidants had a positive effect on renal function but were not significantly associated with kidney stones (33). The limited age range and focus on specific ethnic groups in these studies require further investigation and verification. Given the controversial evidence mentioned above, our study is necessary and important.

Oxidative stress (OS), which arises from an imbalance between reactive oxygen species (ROS) and the body’s antioxidant defenses, significantly contributes to the formation of kidney stones (34). Under normal conditions, ROS perform essential functions such as signaling molecules, mediating cell growth, and immune responses. However, excessive production of ROS can damage biomolecules, triggering inflammatory responses and sustained renal tubular damage, ultimately facilitating the formation, growth, and aggregation of stone crystals (35). Hong et al. found that dietary polyphenols, a potent class of natural antioxidants, can modulate the expression and activity of endogenous antioxidant enzymes, influence OS-related signaling pathways, and maintain cellular morphology and functionality (36). Studies have shown that vitamins E and C can attenuate ROS production and protect renal epithelial cells from oxalate-induced oxidative damage, with synergistic effects observed when combined (15). Including foods rich in antioxidants in the daily diet may, therefore, help to reduce the prevalence and recurrence of kidney stones.

Similarly, changes in urine pH are identified as another critical factor in the formation of kidney stones. Uric acid tends to crystallize and form stones when the urine pH drops below 5.5, increasing the likelihood of uric acid stones (37–39). Certain antioxidants can alter the chemical properties of urine; for example, vitamin A can elevate urinary pH and enhance citrate excretion, thereby decreasing the potential for stone formation (12, 40). In addition, antioxidants can directly or indirectly affect the nucleation, growth, and aggregation of crystals in the urine, reducing the risk of stone formation by reducing crystal formation or promoting crystal dissolution (41). Animal studies have shown that antioxidants such as quercetin, vitamin E, and taurine can reduce crystal deposition in rat models of hyperoxaluria-induced kidney stones, consistent with the above findings (42).

Thirdly, inflammatory immune responses are closely linked to the formation of kidney stones (43). The excessive production of free radicals activates several inflammatory cells and cytokines, increasing cytokines such as TNFα, IL-1β, IL-8, and IL-10 (44). This, in turn, damages renal tubular epithelial cells and causes calcium oxalate crystals to adhere, facilitating stone formation in the papillary and medullary regions of the kidney (45). Antioxidants can reduce inflammatory responses by neutralizing free radicals. In addition, antioxidants may inhibit kidney stone formation by suppressing inflammatory mediator production by inhibiting NF-kB and MAPKs pathways (46). Oxidative stress and inflammatory responses are mutually reinforcing in kidney stone formation, and antioxidants control the occurrence of kidney stones by inhibiting these two processes. For more details on the possible mechanisms by which antioxidants may counteract kidney stone formation, please refer to Table 3.

**Table 3:** Animal experimental and clinical evidence of the impact of antioxidants on kidney stone formation.

Antioxidants	Study type	Study design	Possible mechanisms	Ref.
Vitamin A	Animal Model	Male Wistar rats induced with hyperoxaluria (oral 0.5% ethylene glycol)	Urine pH ↑ citrate excretion ↑	(12)
	Clinical Participants	Vitamin A-deficient boys administered 24,000 IU of retinyl palmitate daily	Inhibitory activity on calcium oxalate crystal growth ↑ excretion of calcium and oxalate ↓	(47)
Vitamin C	*In Vitro* Experiment	Evaluation of the effects of physiological urinary oxalate concentrations on normal and antioxidant-deficient LLC-PK1 cell cultures	ROS production ↓ oxalate-induced tubular oxidative damage ↓	(15)
Vitamin E	Animal Model	Male Sprague–Dawley rats induced with hyperoxaluria (oral 150 mg ethylene glycol)	Oxidative stress damage ↓ calcium oxalate crystal deposition ↓	(48)
	Animal Model	Male Wistar rats induced with hyperoxaluria (oral 0.75% ethylene glycol)	Tubular epithelial cell death ↓ THP defensive functions ↑ crystal deposition and stone formation ↓	(49)
	Clinical Participants	Patients with calcium oxalate stones (daily oral 400 mg Vitamin E)	THP function restored to normal inhibitory activity on calcium oxalate crystallization ↑	(50)
Zinc	*In Vitro* Experiment	Crystallization of calcium oxalate monohydrate was investigated in the presence of various organic molecules in combination with zinc ions	Chelation with oxalate ions, oxalate ion activity ↓ nucleation rate ↓	(51)
	Animal Model	Male Sprague–Dawley rats induced with calcium oxalate nephrolithiasis (oral 1% ethylene glycol solution)	Inflammatory marker expression ↓ oxalate degradation activity ↑	(52)
Selenium	Animal Model	Male Wistar rats induced with hyperoxaluria (intraperitoneal injection of 0.7% ethylene glycol)	Crystal formation, growth, and aggregation ↓	(53)
	Animal Model	Male dogs induced with calcium oxalate nephrolithiasis (oral 1% ethylene glycol solution)	OPN expression ↓ formation of calcium oxalate stones ↓	(13)
Carotenoids	Animal Model	Male Sprague–Dawley rats induced with calcium oxalate nephrolithiasis (oral 1% ethylene glycol solution)	Oxidative stress damage ↓ inflammation response ↓ deposition of calcium oxalate crystals ↓	(54)

↑ demonstrates increasing trend; ↓ demonstrates decreasing trend. THP (Tamm-Horsfall Protein): A regulatory agent in the nucleation, growth, and aggregation of calcium oxide crystals. OPN (Osteopontin): One of the most crucial components in the matrix of urinary calcium oxalate stones, contributing to the formation of stones. Citrate: Exhibits high affinity for calcium, thereby inhibiting the deposition of calcium crystals in urine. ROS, reactive oxygen species; THP, Tamm-Horsfall protein; OPN, Osteopontin.

There are several strengths to our study. First, it is based on data from NHANES. This database uses a complex sampling design and follows rigorous quality control and standardization procedures to ensure the accuracy and national representativeness of the data. Second, we adjusted for confounding covariates to increase the reliability of our results. In addition, subgroup analyses and sensitivity analyses have demonstrated the robustness of our findings. However, our study is subject to several limitations. First, the cross-sectional nature of our design prevents us from determining causal relationships between CDAI levels and the prevalence and recurrence of kidney stones. Second, antioxidant intake was derived from two 24 h recall interviews, subject to recall bias. Third, due to limitations in the NHANES database, we lack data on the composition of kidney stones and cannot precisely record and analyze the types of water intake and specific foods consumed by the subjects. This restricts our analysis of the relationship between CDAI levels and various types of kidney stones and may affect our comprehensive assessment of the factors associated with kidney stone formation. Fourth, although we controlled for several conventional variables, the influence of all potential confounders could not be completely excluded. Finally, our study population consisted of American adults, which may affect the generalizability of our results.

## 5 Conclusion

Our findings suggest an association between increased intake of specific dietary antioxidants and reduced prevalence and recurrence of kidney stones, highlighting the potential role of these nutrients in reducing such occurrences. These insights could inform prevention and treatment strategies for kidney stones. However, given the variability in individual antioxidant responses and the complexity of dietary factors, additional prospective studies and foundational research are required to confirm these associations and elucidate the underlying mechanisms.

## Data availability statement

Publicly available datasets were analyzed in this study. This data can be found at: detailed information about this study can be found at the NHANES online website: https://www.cdc.gov/nchs/nhanes/index.htm.

## Ethics statement

The studies involving humans were approved by NCHS Research Ethics Review Board, which is affiliated with the National Center for Health Statistics (NCHS). The studies were conducted in accordance with the local legislation and institutional requirements. Written informed consent for participation was not required from the participants or the participants’ legal guardians/next of kin in accordance with the national legislation and institutional requirements.

## Author contributions

HZ: Writing – original draft, Conceptualization, Data curation, Formal analysis. YC: Data curation, Formal analysis, Writing – original draft, Writing – review & editing. YF: Conceptualization, Writing – review & editing. HC: Conceptualization, Data curation, Formal analysis, Writing – original draft.
